# 11979 Using whole-exome and mtDNA sequencing to develop a testing algorithm for diagnosis of mitochondrial disease in Puerto Ricans

**DOI:** 10.1017/cts.2021.671

**Published:** 2021-03-30

**Authors:** Elinette Albino, Carmen Buxo, Fernando Scaglia, Alberto Santiago-Cornier

**Affiliations:** 1 University of Puerto Rico-Medical Sciences Campus; 2 Baylor College of Medicine; 3 Ponce Health Sciences University

## Abstract

ABSTRACT IMPACT: Alterations in mitochondrial metabolism affect any tissue, especially those with the highest demand for energy. As the symptoms and clinical manifestations are heterogenous, disease diagnosis is challenging. The implementation of genetic-first approach in the diagnosis of mitochondrial diseases will expedite confirmation, treatment, management, and counseling of affected Puerto Rican individuals. OBJECTIVES/GOALS: Mitochondrial diseases are rare, and diagnosis is complex due to the heterogeneity of clinical manifestations. We aim to develop and implement a testing algorithm using a genetics-first approach, facilitating the identification of variants that contribute to mitochondrial disease’s etiology and influence onset and progression in Puerto Ricans. METHODS/STUDY POPULATION: This is a cross-sectional study for characterizing clinical laboratory results from profiles used to evaluate metabolic diseases in individuals with suspected mitochondrial disorders from 2018 to 2021. A subset of 25 individuals from biochemical profile will be recruited to analyze their medical and family history, metabolic biomarkers in blood and urine, hearing test, imaging and chromosomal microarray. The implementation of a genetic testing algorithm using whole exome and mitochondrial DNA sequencing will be performed in a subset of 11 randomized individuals. Descriptive analysis will be reported, including a catalog of all variants. Multivariate analysis will be performed to estimate the statistical association between variants and phenotypes reported and adjusting for potential confounders. RESULTS/ANTICIPATED RESULTS: The biochemical profile of pediatric Puerto Rican individuals suspected of having mitochondrial diseases will be altered and can be used to differentiate among other metabolic causes. We expect to find altered levels of lactate, pyruvate and carnitines in serum, as well as altered organic acids in urine. The implementation of a testing algorithm using both, mitochondrial DNA and whole exome sequencing as first approach will be enabling the identification of disease-causing variants, thus enhancing and confirming the diagnosis of mitochondrial disease in Puerto Ricans. We will be able to identify rare/novel variants specific to our Hispanic population, for both nuclear and mitochondrial DNA. DISCUSSION/SIGNIFICANCE OF FINDINGS: This study will help to characterize the metabolic profile of pediatric Puerto Ricans. No previous study has been reported that describes testing algorithms for genetic diagnosis of mitochondrial disease in our population. Variants found will contribute to a deep understanding of the genetic contribution to phenotypes and disease susceptibility.

